# iTRAQ-Based Quantitative Proteomics Reveals the Energy Metabolism Alterations Induced by Chlorogenic Acid in HepG2 Cells

**DOI:** 10.3390/nu14081676

**Published:** 2022-04-18

**Authors:** Shoko Takahashi, Kenji Saito, Xuguang Li, Huijuan Jia, Hisanori Kato

**Affiliations:** Health Nutrition, Department of Applied Biological Chemistry, Graduate School of Agricultural and Life Sciences, The University of Tokyo, 1-1-1 Yayoi, Bunkyo-ku, Tokyo 113-8657, Japan; takahashi@genequest.jp (S.T.); skkj774@gmail.com (K.S.); lixuguang1225@gmail.com (X.L.); akatoq@g.ecc.u-tokyo.ac.jp (H.K.)

**Keywords:** coffee, chlorogenic acid, energy metabolism, lipid metabolism, HepG2 cells, proteomics

## Abstract

Epidemiological studies have suggested that coffee consumption is associated with a decrease in the risk of developing obesity and diabetes; however, the detailed mechanisms underlying these effects of coffee consumption remain poorly understood. In this study, we examined the effects of chlorogenic acid on energy metabolism in vitro. Hepatocellular carcinoma G2 (HepG2) cells were cultured in a medium containing chlorogenic acid. Chlorogenic acid increased the activity of mitochondrial enzymes, including citrate synthase, isocitrate dehydrogenase, and malate dehydrogenase (MDH), which are involved in the tricarboxylic acid (TCA) cycle. Proteome analysis using the isobaric tags for the relative and absolute quantitation (iTRAQ) method revealed the upregulation of proteins involved in the glycolytic system, electron transport system, and ATP synthesis in mitochondria. Therefore, we propose a notable mechanism whereby chlorogenic acid enhances energy metabolism, including the TCA cycle, glycolytic system, electron transport, and ATP synthesis. This mechanism provides important insights into understanding the beneficial effects of coffee consumption.

## 1. Introduction

Coffee has been consumed by humans for thousands of years and is currently one of the most popular beverages worldwide. Accumulating epidemiological evidence suggests that coffee, as a functional food, plays a role in the prevention of diseases. Therefore, understanding the effects of coffee intake on health is desirable. Lines of evidence, including those from epidemiological studies, have suggested that the intake of coffee may reduce the risk of developing obesity and diabetes [1,2]. A meta-analysis of prospective cohort research [3] revealed that the intake of coffee ingredients helped decrease the risk of type 2 diabetes. However, the detailed mechanisms underlying the beneficial effects of coffee consumption are still poorly understood.

Many studies on the effects of coffee have focused on liver tissue, including a meta-analysis of four cohort studies by Larsson and Wolk [4], which revealed that consuming two cups of coffee per day decreased the risk of hepatic cancer by 43%. The effects of coffee ingredients on hepatic cells have been examined in several studies, some of which used human hepatocellular carcinoma G2 (HepG2) cells to explore the physiological action of coffee ingredients. HepG2 cells are considered a feasible model for in vitro analysis of xenobiotic metabolism and hepatotoxicity since they perform the majority of the physiological functions like normal human hepatocytes [5,6]. For example, Riedel et al. [7] studied the effects of trigonelline on hepatic glucose metabolism, while Vaynshteyn and Jeong [8] and Kalthoff et al. [9] reported the effects of caffeine on the expression of cytochrome P450 1A2 (CYP1A2) and glucuronosyltransferase in HepG2 cells, respectively. Since coffee ingredients and/or their metabolites remain in the blood after the coffee is consumed, the findings of these in vitro studies using coffee ingredients could shed some light on the bioactivity of coffee. As a model of metabolically active cells, HepG2 was also used in this experiment.

Our previous in vivo studies, conducted with multi-omics analyses (transcriptomics, proteomics, and metabolomics), suggested that coffee intake activates the urea cycle and the tricarboxylic acid (TCA) cycle in the livers of C57BL/6J mice fed a high-fat diet [10,11]. To the best of our knowledge, there have been no studies on the effects of coffee ingredients and hepatic energy metabolism in cultured hepatic cells. In the present study, we explored the effects of coffee and chlorogenic acid (CGA) on energy metabolism in an in vitro experiment using HepG2 cells.

## 2. Materials and Methods

### 2.1. Cell Culture

Human hepatocyte HepG2 cells (American Type Culture Collection, Rockville, MD, USA) were cultured in Dulbecco’s Modified Eagle’s Medium (DMEM)-D5796 (Sigma-Aldrich, Tokyo, Japan) containing 10% fetal bovine serum (FBS) (Nichirei Bioscience, Tokyo, Japan) and 1% 100 U/mL penicillin/streptomycin (Sigma-Aldrich, Tokyo, Japan). Each assay described below was performed by culturing approximately 2.5 × 10^5^ cells/well seeded in 24-well plates for 48 h. HepG2 cells were cultured at 37 °C with 5% CO_2_.

### 2.2. Coffee Ingredients

Caffeinated coffee powder (Goldblend, Nestle Japan, Hyogo, Japan), decaffeinated coffee powder (Goldblend, Nestle, Hyogo, Japan), and CGA (Sigma-Aldrich, Tokyo, Japan) were used for the experiments. HepG2 cells were cultured in DMEM containing these ingredients (50, 100, and 200 μg/mL).

### 2.3. MTT (3-[4,5-Dimethylthiazol-2-yl]-2,5-diphenyltetrazolium bromide) Assay

HepG2 cells were cultured in DMEM containing coffee ingredients for 24 h, and after being washed with phosphate-buffered saline (PBS), they were incubated at 37 °C for 3 h in DMEM containing 0.5 mg/mL of MTT solution M5655 (thiazolyl blue tetrazolium bromide, Sigma-Aldrich, Tokyo, Japan). The MTT solution was removed and 0.04 N HCl/isopropanol was added, and 5 min later, the absorbance of the supernatant was measured (OD at 540 nm).

### 2.4. Enzyme Activity Measurement

Spectrophotometric analysis of the enzymes was performed as described [12,13]. Briefly, citrate synthase (CS): The cell solution was mixed with 100 mM Tris-HCl (pH 8.0), 10 mM DTNB (5,5′-dithio-bis-(2-nitrobenzoic acid)), and 2.5 mM acetyl-CoA, and the absorbance was measured using Biolis 24i (Tokyo-Boeki, Tokyo, Japan) (37 °C and 412 nm) after the addition of 50 mM oxaloacetic acid. Isocitrate dehydrogenase (IDH): The cell solution was mixed with 250 mM glycylglycine buffer (pH 7.4), 18 mM manganese chloride, and 20 mM nicotinamide adenine dinucleotide phosphate (NADP), and the absorbance was measured using Biolis 24i (37 °C, 340 nm) after the addition of 6.6 mM DL-isocitric acid. Malate dehydrogenase (MDH): The cell solution was mixed with 100 mM potassium phosphate buffer (pH 7.5) and 0.14 mM β-NADH, and the absorbance was measured using Biolis 24i (37 °C, 340 nm) after the addition of 7.6 mM oxaloacetic acid. Specific activity is defined as units per milligram protein and the data were presented as ratios of the untreated control.

### 2.5. Total RNA Extraction

Total RNA was isolated from HepG2 cells using the TRIzol reagent (Invitrogen Life Technologies, Tokyo, Japan). After culturing the cells under the above conditions, the medium was removed, the cells were washed with PBS, and 500 μL of TRIzol reagent was added and recovered. After standing at room temperature for 5 min, 0.1 mL of chloroform was added and stirred, and the mixture was allowed to stand at room temperature for 2-3 min, then centrifuged at 4 °C and 12,000× *g* for 15 min. An amount of 125 μL of the upper layer was recovered and 250 μL of isopropanol was added. After standing at room temperature for 10 min, it was centrifuged at 12,000× *g* for 10 min at 4 °C. Cells were washed with 75% ethanol, evaporated to dryness and dissolved in 30 µL of RNase-free water. RNA purity was assessed by the ratio of spectrophotometric absorbance at 260 and 280 nm (A260/280 nm) using a NanoDrop ND-1000 spectrophotometer (NanoDrop Products, Wilmington, DE, USA).

### 2.6. Quantitative Real-Time RT-PCR Analysis

Total RNA was used for mRNA analysis by quantitative real-time reverse transcription polymerase chain reaction (RT-PCR). Primers were designed using a web application (PRIMER3), and the sequence information is as follows: since the peroxisome proliferator-activated receptor gamma coactivator 1-alpha (PGC1a) gene has a long sequence, two primers were designed, both near the 3′ end and 5′ end. PGC1a (3′ end) sense: GCAGAGAGGGAACTTTGCAC, antisense: ACAGCCATCAAGAAAGGACA. PGC1a (5′ end) sense: CCTGTGGATGAAGACGGATT, antisense: TGGAGGAAGGACTAGCCTCA. SYBR Green EX (Takara Bio, Madison, WI, USA) was used for the PCR on a real-time PCR detection system (Takara Bio). The relative amounts of mRNA were normalized to glyceraldehyde-3-phosphate dehydrogenase (GAPDH sense: 5′-CGCCTGGAGAAACCTGCCAA-3′, antisense: 5′-GGAGACAACCTGGTCCTCAG-3′) mRNA levels and were expressed as fold-change values.

### 2.7. Proteomics Using Isobaric Tags for Relative and Absolute Quantitation (iTRAQ) Methods and Identification of Regulated Proteins

We performed differential proteomic analysis of HepG2 cells using iTRAQ, as described in our previous study [14]. iTRAQ is a non-gel-based technique used to quantify proteins from different sources in a single experiment. It uses isotope-coded covalent tags. Total protein was extracted using lysis buffer and separated by centrifugation at 12,000× *g* for 30 min at 4 °C. Protein concentrations were determined using the Bradford assay, and pooled proteins (20 μg) for each group (0, 50, 100 and 200 μg/mL of CGA treatment) were subjected to iTRAQ using the iTRAQ^®^ kit, performed according to the manufacturer’s protocol (AB SCIEX, Framingham, MA, USA) with liquid chromatography–tandem mass spectrometry (LC/MS/MS) (TripleTOF TM 5600 + System with Eksigent nanoLC, AB SCIEX). Within an iTRAQ run, differentially changed proteins were determined by ProteinPilot software (AB SCIEX) based on the p-values, which are generated using the peptides used to quantitate the respective protein and give a measure of confidence (95%). In this study, the data of proteins with significantly changed (*p*-value < 0.1) levels were uploaded to the statistical analysis tool Ingenuity Pathway Analysis (IPA), and enrichment analysis was performed [14].

### 2.8. Statistical Analysis

Data were presented as the mean ± standard error (SE). Statistical analysis was performed using a one-way analysis of variance (ANOVA) accompanied by a Tukey’s test. A *p*-value < 0.05 was considered statistically significant.

## 3. Results

### 3.1. The Effect of Coffee Powder and Chlorogenic Acid (CGA) on HepG2 Cell Viability

**Figure 1:**
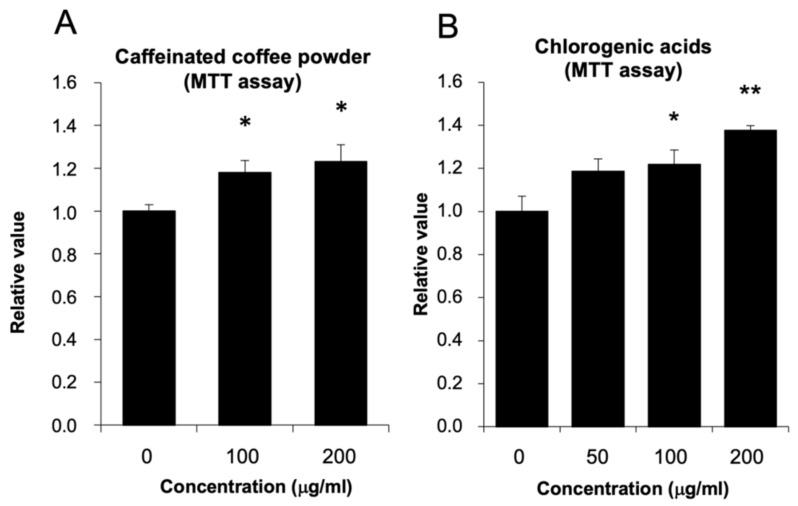
The effects of coffee powder and chlorogenic acid on hepatocellular carcinoma G2 (HepG2) cell viability. HepG2 cells were cultured in Dulbecco’s Modified Eagle’s Medium (DMEM) containing coffee powder or chlorogenic acid for 24 h. The results of the 3-[4,5-Dimethylthiazol-2-yl]-2,5-diphenyltetrazolium bromide (MTT) assay of cells cultured with (**A**) caffeinated coffee powder and (**B**) chlorogenic acid. The data are shown as mean ± SE (*n* = 4). * *p* < 0.05, ** *p* < 0.01 vs. control group.

The potential effects of coffee powder and CGA on the viability and number of HepG2 cells were determined by MTT assay. HepG2 cells were incubated with DMEM containing caffeinated coffee powder, and the results are shown in Figure 1A. HepG2 cells were incubated with caffeinated coffee powder for 24 h, and the cell viability was significantly affected by the caffeinated coffee powder doses used (100 and 200 μg/mL). In addition, CGA also increased the cell viability in a dose-dependent manner (Figure 1B) with the greatest effect at a dose of 200 μg/mL.

### 3.2. Mitochondrial Enzyme Activity Analysis

**Figure 2:**
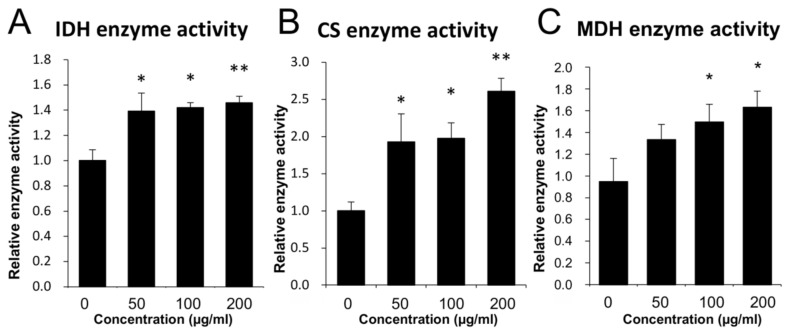
The effects of chlorogenic acid on mitochondrial enzyme activity in HepG2 cells. Confluent cultures of HepG2 cells were cultured with DMEM. Thereafter, different concentrations of chlorogenic acid were added and cells were cultured for 24 h. The enzyme activities of (**A**) isocitrate dehydrogenase (IDH), (**B**) citrate synthase (CS), and (**C**) malate dehydrogenase (MDH) were measured. The data are shown as mean ± SE (*n* = 4). * *p* < 0.05, ** *p* < 0.01 vs. control group.

Since HepG2 cell viability was increased by CGA, we next focused on the effect of CGA on the activities of three enzymes: IDH, CS, and MDH, which are key to the mitochondrial TCA cycle. The results revealed that the activities of all three enzymes were increased by CGA in a concentration-dependent manner (Figure 2). However, CGA addition did not alter the mRNA levels of these enzymes.

### 3.3. Proteomics Using the iTRAQ Method

To explore the effect of CGA on HepG2 cells in a more comprehensive manner, we subjected the proteins extracted from the HepG2 cells treated, as described above, to a proteomic analysis using the iTRAQ method. Using this analysis, 2172 proteins were identified, and 1409 proteins were successfully quantified. Among the identified proteins, 33, 80, and 64 proteins were significantly altered (*p* < 0.1) by CGA at 50, 100, and 200 μg/mL, respectively. Table 1 shows the 64 proteins whose expression was significantly changed by the addition of 200 μg/mL CGA. The data of the proteins whose levels were significantly altered were uploaded to the statistical analysis tool IPA, and the analysis revealed that the differentially expressed proteins were encoded by genes involved in pathways related to the glycolytic system (ALDOA, GAPDH, PGK1, and ENO1) and mitochondrial proteins (NDUFAB1, NDUFAF2, COX5B, ATP5B, and ATP5J). The increase in the levels of a protein involved in the glycolytic system suggests that the signals regulating glucose metabolism and the TCA cycle were enhanced by CGA (Table 1).

## 4. Discussion

The effects of coffee ingredients and coffee polyphenols on cultured hepatic cells have been examined in many studies. First, to evaluate the validity of the study design in which coffee ingredients were directly added to the culture medium, we referenced a previous study that reported the concentrations of coffee ingredients in the blood after the participants drank coffee [15]. The authors reported that the maximum blood concentration of CGA was 5 μM (1.77 μg/mL) after the participants orally ingested 40 g of coffee powder. Another study revealed that after participants drank a cup of coffee, the concentration of the sum of CGA metabolites (including dihydrocaffeic acid and dihydroferulic acid) in their blood was approximately 1 μM [16].

Furthermore, the effect of chlorogenic acid directly added to cultured hepatic cells on lipid metabolism was reported by Liu et al. [17]. In that study, the effects of chlorogenic acid, caffeic acid, and ferulic acid (20–100 μg/mL, respectively) on oleic acid-induced hepatosteatosis were examined, and these coffee polyphenols were found to inhibit the accumulation of lipids in hepatic cells. These studies suggest that coffee polyphenols in the blood could exert physiological actions. Therefore, the present study design in which coffee ingredients were directly added into the culture medium aimed to mimic the conditions after a long-term coffee consumption to some extent.

We performed this study to further investigate the effects of coffee and CGA on energy metabolism in an in vitro experiment using HepG2 cells. Considering the results of the MTT assay, we found that coffee powder and CGA treatment increased cell viability and cell numbers (Figure 1); moreover, our results indicate that CGA at the concentrations tested is not cytotoxic to HepG2 cells, but rather exerts a slight proliferative effect. In addition, PGC1α is reported to be a genetic marker for mitochondrial biogenesis, so we measured its expression by analyzing total RNA extracted from HepG2 cells. The results showed no difference in PGC1α levels with or without the addition of CGA (Figure S1). Therefore, further studies are needed to confirm whether the proliferative effect of CGA is related to mitochondrial activity.

Based on our enzyme activity measurement, CGA increased the activities of three key enzymes, CS, IDH, and MDH (Figure 2). However, the mRNA expression levels of the three genes were not affected by CGA, as observed in the quantitative PCR analysis (data not shown). thus, we speculated that CGA influences the activities of enzymes and not their mRNA levels. Nevertheless, further investigation on the changes in the different isoforms and the time-dependent mRNA expression of the three enzymes, is warranted.

To obtain a more comprehensive view of the mechanism underlying this phenomenon, we performed a proteomic analysis using the iTRAQ method. The enrichment analysis of the proteins whose levels were differentially altered by the addition of 200 μg/mL of CGA revealed that these proteins were either involved in pathways of the glycolytic system (ALDOA, GAPDH, PGK1, and ENO1) or mitochondrial proteins (NDUFAB1, DUFAF2, COX5B, ATP5B, and ATP5J). The increased levels of proteins involved in the glycolytic system suggest that signals between glucose metabolism and the TCA cycle are enhanced by CGA. NADH dehydrogenases (NDUFAB1 and NDUFAF2) are the first enzymes of the electron transport system in mitochondria, and COX5B is an enzyme that is also known to be involved in the electron transport system. ATP synthases (ATP5B, ATP5J) synthesize ATP from the electron transport system in the mitochondria. The increased levels of these mitochondrial proteins suggest that CGA not only enhances the TCA cycle but also other mitochondrial activities, including the electron transport chain and ATP synthesis.

However, additional studies are needed to pinpoint the potential transcriptional factors involved in these processes and clarify the detailed mechanisms impacting the regulation of NADH dehydrogenases and ATP synthases. Although HepG2 cells are widely used to study hepatocyte metabolism as well as hepatocyte physiology, the results presented here should be interpreted with caution owing to their cancerous characteristics. Further studies of primary cell models are needed to confirm the effect of CGA and coffee extract on energy metabolism.

**Figure 3:**
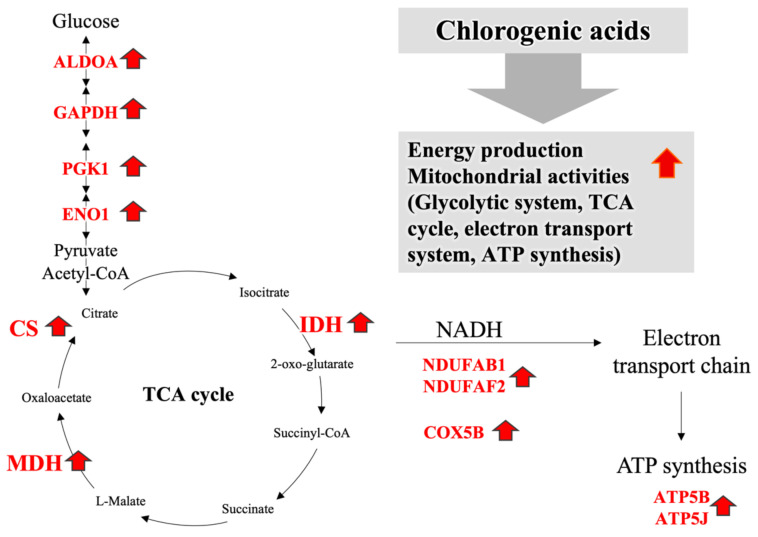
A schematic representation of the proposed mechanism underlying the effects of chlorogenic acid on metabolism as determined by the present in vitro study using HepG2 cells.

Together, the results of the present in vitro study provide novel insights into the mechanism whereby CGA enhances energy metabolism, that is, by increasing hepatic mitochondrial activities, including the TCA cycle, electron transport, and ATP synthesis (Figure 3). Our findings demonstrated that CGA induced hepatic alterations in mitochondrial enzyme activities, and increased expression of proteins involved in the glycolytic system, electron transport system, and ATP synthesis in the mitochondria. This proposed mechanism could be a key aspect in elucidating the beneficial effects of coffee intake.

## 5. Conclusions

In summary, we examined the effects of CGA on energy metabolism in vitro. We propose a mechanism whereby CGA enhances energy metabolism by increasing hepatic mitochondrial activity, including the TCA cycle, glycolysis, electron transport, and ATP synthesis. This study suggests that these alterations provide important insights into the beneficial effects of coffee intake.

## Figures and Tables

**Table 1:** Differentially expressed proteins between control and chlorogenic acid treatment groups by iTRAQ method.

Protein	Name	Protein ID	50 μg/mL		100 μg/mL		200 μg/mL	
			FC	*p*-Value	FC	*p*-Value	FC	*p*-Value
SPTAN1	Isoform 3 of Spectrin alpha chain, brain	Q13813-3	0.93	0.01	0.89	0.00	0.88	0.00
HMGB1	High mobility group protein B1	P09429	0.59	0.00	0.42	0.00	0.55	0.00
AHNAK	Neuroblast differentiation-associated protein AHNAK	Q09666	0.96	0.36	0.83	0.00	0.86	0.00
VSNL1	Visinin-like protein 1	P62760	1.07	0.63	1.27	0.01	1.46	0.00
PSAP	Proactivator polypeptide	P07602	0.95	0.83	1.12	0.11	0.52	0.01
HPCAL1	Hippocalcin-like protein 1	P37235	1.08	0.50	1.10	0.21	1.33	0.01
ZC3H15	Zinc finger CCCH domain-containing protein 15	Q8WU90	0.85	0.19	0.72	0.00	0.82	0.01
GAPDH	Glyceraldehyde-3-phosphate dehydrogenase	P04406	1.30	0.16	1.22	0.00	1.38	0.01
CPLX2	Complexin-2	Q6PUV4	1.34	0.14	1.33	0.01	1.70	0.01
CFL1	Cofilin-1	P23528	0.47	0.01	0.96	0.54	0.66	0.01
PGK1	Phosphoglycerate kinase 1	P00558	1.08	0.43	1.16	0.00	1.21	0.01
KRT18	Keratin, type I cytoskeletal 18	P05783	1.20	0.02	1.17	0.02	1.21	0.02
GLUD1	Glutamate dehydrogenase 1, mitochondrial	P00367	1.08	0.23	1.07	0.12	1.12	0.02
ATP5B	ATP synthase subunit beta, mitochondrial	P06576	1.10	0.51	1.08	0.10	1.18	0.02
IDH1	Isocitrate dehydrogenase [NADP] cytoplasmic	O75874	1.11	0.34	1.16	0.01	1.21	0.03
EIF3A	Eukaryotic translation initiation factor 3 subunit A	Q14152	0.95	0.32	0.96	0.39	0.90	0.03
TPR	Nucleoprotein TPR	P12270	0.99	0.92	0.88	0.06	0.87	0.04
PRDX6	Peroxiredoxin-6	P30041	0.83	0.11	0.96	0.54	0.88	0.04
SRRT	Serrate RNA effector molecule homolog	Q9BXP5	0.79	0.12	0.99	0.95	0.81	0.05
LMO7	LIM domain only protein 7	Q8WWI1	0.97	0.64	0.78	0.01	0.86	0.05
NASP	Nuclear autoantigenic sperm protein	P49321	0.84	0.07	0.87	0.07	0.85	0.05
CALR	Calreticulin	P27797	0.83	0.30	0.87	0.01	0.86	0.05
PNPO	Pyridoxine-5′-phosphate oxidase	Q9NVS9	1.06	0.60	1.11	0.15	1.17	0.05
HSPA5	78 kDa glucose-regulated protein	P11021	1.24	0.01	1.04	0.36	1.12	0.05
CALD1	Isoform HELA L-CAD II of Caldesmon	Q05682-5	0.81	0.04	0.75	0.02	0.82	0.05
PTGES3	Prostaglandin E synthase 3	Q15185	0.36	0.07	0.92	0.78	0.67	0.05
VDAC1	Voltage-dependent anion-selective channel protein 1	P21796	1.20	0.14	1.22	0.11	1.32	0.05
WDR1	WD repeat-containing protein 1	O75083	1.11	0.38	1.14	0.05	1.19	0.05
ENO1	Alpha-enolase	P06733	1.26	0.14	1.17	0.02	1.28	0.05
ATIC	Bifunctional purine biosynthesis protein PURH	P31939	1.09	0.31	1.10	0.18	1.14	0.06
RPLP2	60S acidic ribosomal protein P2	P05387	1.37	0.22	1.10	0.27	1.50	0.06
HSPA9	Stress-70 protein, mitochondrial	P38646	0.85	0.22	0.91	0.30	0.85	0.06
MYL6	Myosin light polypeptide 6	P60660	1.12	0.22	1.09	0.34	1.24	0.06
FKBP4	Peptidyl-prolyl cis-trans isomerase FKBP4	Q02790	1.16	0.25	1.11	0.33	1.23	0.06
RPL23A	60S ribosomal protein L23a	P62750	0.70	0.21	0.63	0.01	0.68	0.06
TPD52L2	Tumor protein D54	O43399	0.87	0.27	0.75	0.02	0.82	0.06
NIPSNAP3A	Protein NipSnap homolog 3A	Q9UFN0	0.82	0.17	1.08	0.34	1.20	0.06
TPM3	Isoform TM30nm of Tropomyosin alpha-3 chain	P06753-2	1.28	0.29	1.09	0.35	1.45	0.06
NDUFAB1	Acyl carrier protein, mitochondrial	O14561	1.35	0.33	1.30	0.11	1.87	0.06
CYCS	Cytochrome c	P99999	0.73	0.03	0.69	0.02	0.80	0.07
PDLIM5	PDZ and LIM domain protein 5	Q96HC4	0.83	0.02	0.81	0.02	0.87	0.07
ANPEP	Aminopeptidase N	P15144	0.92	0.13	0.91	0.09	0.90	0.07
ADK	Adenosine kinase	P55263	0.81	0.15	0.91	0.36	0.79	0.07
ATP5J	ATP synthase-coupling factor 6, mitochondrial	P18859	2.10	0.12	1.46	0.23	2.29	0.08
PCBD1	Pterin-4-alpha-carbinolamine dehydratase	P61457	1.10	0.54	1.12	0.25	1.37	0.08
UGDH	UDP-glucose 6-dehydrogenase	O60701	1.10	0.38	1.10	0.08	1.15	0.08
NDUFAF2	Mimitin, mitochondrial	Q8N183	1.18	0.22	1.11	0.37	1.31	0.08
TTC1	Tetratricopeptide repeat protein 1	Q99614	1.01	0.89	1.05	0.56	1.17	0.09
LASP1	LIM and SH3 domain protein 1	Q14847	0.94	0.60	0.98	0.82	1.25	0.09
ALDOA	Fructose-bisphosphate aldolase A	P04075	1.27	0.05	1.34	0.04	1.35	0.09
FTH1	Ferritin heavy chain	P02794	0.59	0.09	0.65	0.08	0.70	0.09
ACAA2	3-ketoacyl-CoA thiolase, mitochondrial	P42765	1.18	0.11	1.13	0.34	1.21	0.09
ENSA	Isoform 8 of Alpha-endosulfine	O43768-8	2.26	0.19	1.57	0.17	2.77	0.09
BAT1	Spliceosome RNA helicase BAT1	Q13838	0.76	0.22	1.04	0.62	0.76	0.09
RPL13	60S ribosomal protein L13	P26373	1.24	0.22	1.16	0.68	1.24	0.09
UGGT1	UDP-glucose:glycoprotein glucosyltransferase 1	Q9NYU2	1.00	0.99	0.83	0.06	0.88	0.09
UBQLN1	Ubiquilin-1	Q9UMX0	1.11	0.28	1.13	0.22	1.21	0.10
MESDC2	LDLR chaperone MESD	Q14696	0.83	0.12	0.72	0.06	0.82	0.10
NUTF2	Nuclear transport factor 2	P61970	1.37	0.05	1.58	0.01	1.42	0.10
STRAP	Serine-threonine kinase receptor-associated protein	Q9Y3F4	1.16	0.12	1.25	0.04	1.18	0.10
COX5B	Cytochrome c oxidase subunit 5B, mitochondrial	P10606	1.09	0.75	1.06	0.61	1.34	0.10

Differentially expressed proteins (*p* < 0.1) were treated with 50, 100, and 200 μg/mL of chlorogenic acid. FC: fold change. iTRAQ: isobaric tags for relative and absolute quantitation.

## Data Availability

The data presented in this study are available on request from the corresponding author.

## Supplementary Materials

The following supporting information can be downloaded at https://www.mdpi.com/article/10.3390/nu14081676/s1, Figure S1: The effects of chlorogenic acid on the mRNA expression of *PGC1a*. HepG2 cells were cultured in DMEM containing chlorogenic acid (200 μg/mL), and the mRNA expression at each time point was measured by RT-PCR. **A**: Using the primer designed near the 3′ end. **B**: Using the primer designed near the 5′ end.

## References

[1] Van Dam R.M., Hu F.B. (2005). Coffee consumption and risk of type 2 diabetes: A systematic review. JAMA.

[2] Carlsson S., Hammar N., Grill V., Kaprio J. (2004). Coffee consumption and risk of type 2 diabetes in Finnish twins. Int. J. Epidemiol..

[3] Huxley R., Lee C.M., Barzi F., Timmermeister L., Czernichow S., Perkovic V., Grobbee D.E., Batty D., Woodward M. (2009). Coffee, decaffeinated coffee, and tea consumption in relation to incident type 2 diabetes mellitus: A systematic review with meta-analysis. Arch. Intern. Med..

[4] Larsson S.C., Wolk A. (2007). Coffee consumption and risk of liver cancer: A meta-analysis. Gastroenterology.

[5] Li D., Na X., Wang H., Wang C., Yuan Z., Zhu B.W., Tan M. (2020). The effects of carbon dots produced by the Maillard reaction on the HepG2 cell substance and energy metabolism. Food Funct..

[6] Vijayakumar K., Rengarajan R.L., Radhakrishnan R., Mathew S., Qadri I., Vijaya Anand A. (2019). Psidium guajava Leaf Extracts and Their Quercetin Protect HepG2 Cell Lines Against CCL4 Induced Cytotoxicity. Indian J. Clin. Biochem..

[7] Riedel A., Hochkogler C.M., Lang R., Bytof G., Lantz I., Hofmann T., Somoza V. (2014). N-methylpyridinium, a degradation product of trigonelline upon coffee roasting, stimulates respiratory activity and promotes glucose utilization in HepG2 cells. Food Funct..

[8] Vaynshteyn D., Jeong H. (2012). Caffeine induces CYP1A2 expression in rat hepatocytes but not in human hepatocytes. Drug Metab. Lett..

[9] Kalthoff S., Ehmer U., Freiberg N., Manns M.P., Strassburg C.P. (2010). Coffee induces expression of glucuronosyltransferases by the aryl hydrocarbon receptor and Nrf2 in liver and stomach. Gastroenterology.

[10] Takahashi S., Saito K., Jia H., Kato H. (2014). An integrated multi-omics study revealed metabolic alterations underlying the effects of coffee consumption. PLoS ONE.

[11] Takahashi S., Egashira K., Saito K., Jia H.J., Abe K., Kato H. (2014). Coffee intake down-regulates the hepatic gene expression of peroxisome proliferator-activated receptor gamma in C57BL/6J mice fed a high-fat diet. J. Funct. Foods.

[12] Sauer S.W., Okun J.G., Schwab M.A., Crnic L.R., Hoffmann G.F., Goodman S.I., Koeller D.M., Kolker S. (2005). Bioenergetics in glutaryl-coenzyme A dehydrogenase deficiency: A role for glutaryl-coenzyme A. J. Biol. Chem..

[13] Shepherd D., Garland P.B. (1969). [2] Citrate synthase from rat liver: [EC 4.1.3.7 Citrate oxaloacetage-lyase (CoA-acetylating)]. Methods in Enzymology.

[14] Jia H., Wen Y., Aw W., Saito K., Kato H. (2021). Ameliorating Effects of Coriander on Gastrocnemius Muscles Undergoing Precachexia in a Rat Model of Rheumatoid Arthritis: A Proteomics Analysis. Nutrients.

[15] Monteiro M., Farah A., Perrone D., Trugo L.C., Donangelo C. (2007). Chlorogenic acid compounds from coffee are differentially absorbed and metabolized in humans. J. Nutr..

[16] Williamson G., Dionisi F., Renouf M. (2011). Flavanols from green tea and phenolic acids from coffee: Critical quantitative evaluation of the pharmacokinetic data in humans after consumption of single doses of beverages. Mol. Nutr. Food Res..

[17] Liu Y., Wang D., Zhang D., Lv Y., Wei Y., Wu W., Zhou F., Tang M., Mao T., Li M. (2011). Inhibitory effect of blueberry polyphenolic compounds on oleic acid-induced hepatic steatosis in vitro. J. Agric. Food Chem..

