# Supplementary material for: iTRAQ-Based Quantitative Proteomics Reveals the Energy Metabolism Alterations Induced by Chlorogenic Acid in HepG2 Cells
Source: Nutrients. 2022 Apr 18;14(8):1676. doi: 10.3390/nu14081676 (PMC9032979; doi:10.3390/nu14081676)
Supplement: Supplementary file 1 [file nutrients-14-01676-s001.zip › nutrients-1633696-supplementary.pdf]

Supplementary Figure and Figure legend

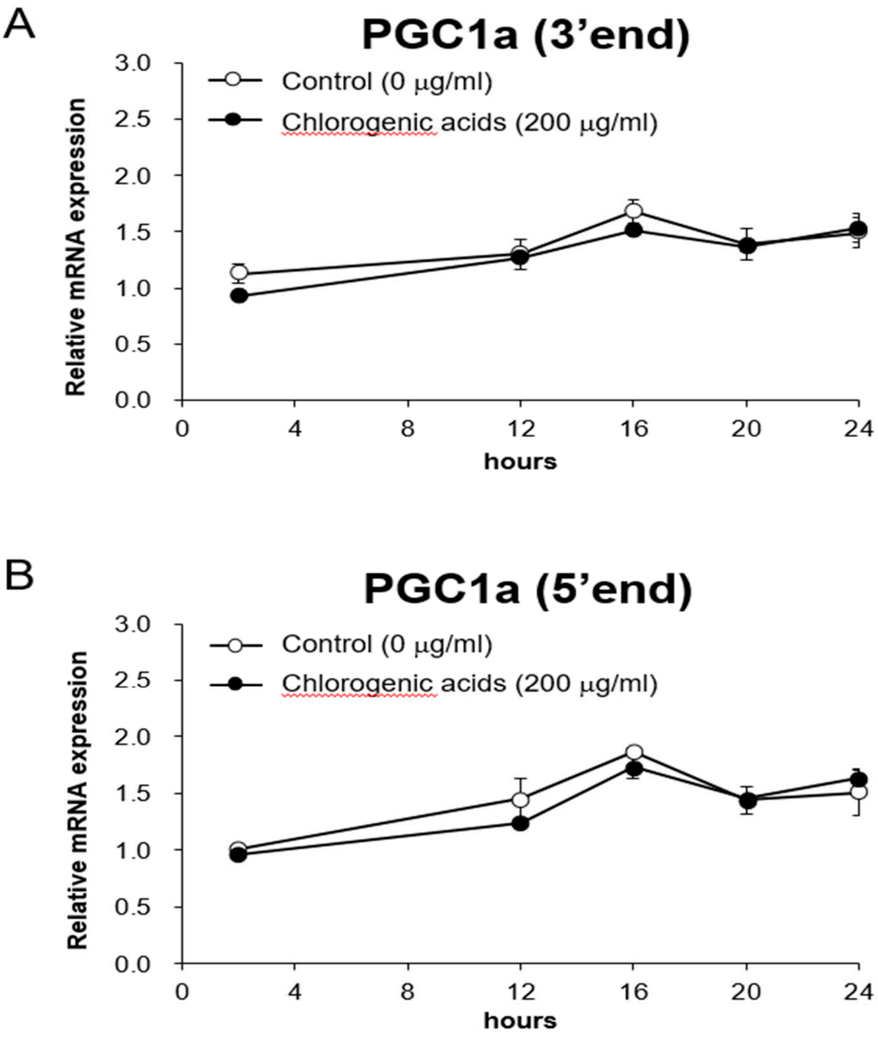

Figure S1. The effects of chlorogenic acid on the mRNA expression of PGC1a gene. HepG2 cells were cultured in DMEM medium containing chlorogenic acid (200 μg/ml), and the mRNA expression at each time point was measured by RT-PCR. **A**: Using the primer designed near the 3' end. **B**: Using the primer designed near the 5' end. The data are shown as mean ± SE (*n* = 4).
